# Cyanidin-3-glucoside suppresses the progression of lung adenocarcinoma by downregulating TP53I3 and inhibiting PI3K/AKT/mTOR pathway

**DOI:** 10.1186/s12957-021-02339-7

**Published:** 2021-08-06

**Authors:** Xiaojun Chen, Weixia Zhang, Xiuzhen Xu

**Affiliations:** 1 Department of Emergency, Weifang Hospital of Traditional Chinese Medicine, Weifang, Shandong 261041 P.R. China; 2 Department of Urology, Weifang Hospital of Traditional Chinese Medicine, Weifang, Shandong 261041 P.R. China; 3 Department of Emergency, Weifang Brain Hospital, 423 Dongfeng West Street, Weicheng District, Weifang, Shandong 261021 P.R. China

**Keywords:** LUAD, C3G, TP53I3, Apoptosis, Metastasis, PI3K/AKT/mTOR pathway

## Abstract

**Background:**

The aim of this study is to unravel the role of Cyanidin-3-glucoside (C3G) and its potential mechanisms in lung adenocarcinoma (LUAD).

**Methods:**

The cell clones, proliferation, apoptosis, migration, and invasion in H1299 and A549 cells were determined by colony formation assay, 5-ethynyl-20 deoxyuridine (EdU) assay, flow cytometry, and transwell assay, respectively. The expression of p53-induced gene 3 (TP53I3) was assessed and the prognostic values of TP53I3 in LUAD via the dataset from the Cancer Genome Atlas (TCGA). In addition, the mRNA and protein expressions were detected by quantitative real-time PCR (qRT-PCR) and western blot.

**Results:**

C3G inhibited the proliferation, migration, and invasion of, and also promoted the apoptosis in H1299 and A549 cells. The database of TCGA showed TP53I3 was highly expressed in LUAD tissues and correlated with the poor prognosis of LUAD patients. Moreover, we also found that C3G inhibited the proliferation, migration and invasion, and promoted apoptosis in H1299 and A549 cells by downregulating TP53I3. Additionally, C3G could inhibit the activation of phosphatidylinositol 3′-kinase (PI3K)/protein kinase B (AKT)/mammalian target of rapamycin (mTOR) pathway in H1299 and A549 cells by downregulating TP53I3.

**Conclusion:**

This study demonstrated that C3G could inhibit the proliferation, migration and invasion, and also facilitate the apoptosis through downregulating TP53I3 and inhibiting PI3K/AKT/mTOR pathway in LUAD.

## Background

Lung cancer is the second most frequent cancer in both men and women, and it is considered to be one of the major causes for cancer-related death worldwide [1, 2]. Non-small cell lung cancer (NSCLC) accounts for about 85% of diagnosed lung cancer cases [1, 3, 4]. Moreover, NSCLC can be divided into lung adenocarcinoma (LUAD), lung squamous cell carcinoma, and large cell cancer [5]. Despite the recent advances in surgery, radiotherapy, and chemotherapy, the 5-year survival rates remain dismal [6]. Unfortunately, most patients are usually diagnosed at an advanced stage [7]. Therefore, exploring new drugs and therapeutic targets for LUAD is urgently needed.

Cyanidin-3-glucoside (C3G), one of the most abundant anthocyanidin in a wide variety of fruits and vegetables, has been reported to have multiple beneficial effects for cardiovascular diseases, diabetes, and inflammation [8–10]. In recent years, some studies have indicated that C3G can exert inhibitory effects in a variety of cancers. For example, C3G could attenuate the migratory and invasive capacity in breast cancer cells by regulating Sirt1 expression [11]. Moreover, the study by Chen et al. [12] has reported that C3G can exhibit an inhibitory effect on the migration and invasion of lung cancer cells. However, the potential mechanism of C3G on LUAD has not been widely explored.

The p53-induced gene 3 (TP53I3 or PIG3), one of the p53-induced genes, is involved in both the p53-mediated apoptosis and DNA damage response [13–15]. TP53I3 is reported to play important roles in apoptosis via reactive oxygen species generation and oxidative stress induction [13]. In other words, TP53I3 alone cannot directly induce apoptosis unless it cooperates with a set of simultaneously activated pro-apoptotic genes induced by reactive oxygen species [13]. However, the roles of TP53I3 alone on apoptosis are rarely explored in previous studies, and investigated in this study. In recent years, the function of TP53I3 in tumors has attracted much attention due to its relationship with apoptosis and DNA damage response. Xu et al. [16] have reported that TP53I3 could facilitate the growth of papillary thyroid cancer. A study by Park et al. has confirmed that the downregulation of TP53I3 can inhibit the migration and invasion of colon cancer cells [17]. Gu et al. [18] also suggest that TP53I3 can promote cell migration and invasion in LUAD. UALCAN database suggests that TP53I3 was highly expressed in LUAD patients’ tissues, and LUAD patients with higher TP53I3 levels had a worse prognosis, which confirms the study of Gu et al. [18]. Additionally, the bioinformatics analysis shows that C3G is associated with TP53I3 in LUAD. Therefore, whether C3G exerts effects on LUAD cells by regulating TP53I3 was investigated.

In the present study, the findings revealed that C3G could inhibit the proliferation, migration, and invasion, and also facilitate the apoptosis by downregulating TP53I3 in LUAD. Moreover, C3G could inhibit the activation of PI3K/AKT/mTOR pathway by downregulating TP53I3 in LUAD cells. The findings of this study suggest that C3G is a potential compound for LUAD treatment.

## Methods

### Cell culture and reagents

Human LUAD cell lines (H1299 and A549) and normal lung epithelial cell line BEAS-2B were supplied by American Type Culture Collection (ATCC, USA). All cell lines were cultured at 37 °C in a humidified atmosphere containing 5% CO_2_ in Dulbecco’s modified Eagle medium (DMEM, Invitrogen, USA) supplemented with 10% fetal bovine serum (FBS, Wisent, Canada). C3G (98.0% purity) was obtained from Tokiwa Phytochemical Co., Ltd., Japan, and dissolved in dimethyl sulfoxide (DMSO, Sigma-Aldrich, USA) for the subsequent assays.

### Cell transfection

Small interfering RNA targeting TP53I3 (si-TP53I3) and siRNA negative control (si-NC) were purchased from GenePharma (Shanghai, China). In brief, H1299 and A549 cells (6 × 10^5^/well) were planted into a 6-well plate and then cultured with or without C3G for 24 h. Then, the cells were transfected with si-TP53I3 or si-NC by using Lipofectamine 3000 (Invitrogen, USA) following the manufacturer’s instructions. Forty-eight hours later, the cells were harvested for the subsequent experiments.

### Cell counting kit-8 assay

Cell viability was assessed with Cell Counting Kit-8 (CCK-8, Dojindo Molecular Technologies, USA). Simply, H1299, A549, and BEAS-2B cells (3 × 10^3^/well) were seeded in 96-well plates and then exposed to different concentrations of C3G (0, 5, 10, 20, 40, and 80 μM) for 24 h. After that, the cells were harvested, and then CCK-8 solution (10 μL) was added to each well. Finally, the absorbance at 450 nm was tested by a microplate reader (model 680, Bio-Rad, USA).

### Colony formation assay

H1299 and A549 cells were exposed to C3G (10, 20, and 40 μM) for 24 h. Afterwards, they were seeded in 12-well plates in triplicate and cultured for 2 weeks at 37 °C in a 5% CO_2_ incubator. Culture medium was replaced every 3 days. Then, 4% paraformaldehyde was applied to fix the colonies. Finally, the cell colonies were stained with 0.1% crystal violet (Beyotime, Jiangsu, China) for 20 min and counted under a light microscopy.

### 5-Ethynyl-20-deoxyuridine (EdU) assay

The proliferation of H1299 and A549 cells was determined by using the EdU incorporation assay kit (Ribobio, Guangzhou, China). Simply, the cells were cultured with EdU solution for 2 h and then fixed with PBS containing 4% paraformaldehyde. Subsequently, the nucleus was stained with 4′,6-diamidino-2-phenylindole (DAPI, Beyotime Biotechnology, Shanghai, China). Finally, the cells were visualized with a fluorescence microscopy.

### Flow cytometry analyses

To investigate cell apoptosis, flow cytometry was carried out. The cells were resuspended and stained with Annexin V-FITC for 5 min in dark at 4 °C under the construction of the Annexin V-propidium iodide (PI) kit (Beyotime Biotechnology, Shanghai, China). Subsequently, the cells were stained with propidium iodide (PI) for 5 min, and finally analyzed using a FACScan flow cytometer (BD Biosciences, USA).

### Transwell assay

Cell invasion assay was carried out by using a Corning Polycarbonate Membrane Insert transwell chamber (8-μm pore size, Corning, USA) coated with Matrigel (BD Bioscience, USA) and cell migration assay was performed in a similar method without Matrigel. The cells with 200 μL FBS-free medium were placed into the upper chamber and 600 μL medium containing 20% FBS was added into the lower chamber. After a 24-h incubation, the non-migrated or non-invaded cells were removed with a cotton swab, while the migrated or invaded cells were fixed with 4% paraformaldehyde for 30 min and stained with 0.5% crystal violet for 30 min. Finally, the migrated or invaded cell numbers were counted under microscope.

### qRT-PCR analysis

Total RNA was extracted by using TRIzol Reagent (Thermo Fisher, USA), followed by reverse-transcription for complementary DNA (cDNA) synthesis with the One Step PrimeScript cDNA Synthesis Kit (Takara, Japan). Then, qRT-PCR analysis was performed on ABI 7500 system (Applied Biosystem, USA) with the SYBR Premix Ex Taq II kit (Takara, Japan). GAPDH was considered as the endogenous control. And the relative expression levels were calculated by the 2^−ΔΔCt^ method. All primers were designed by GenePharma (Shanghai, China) as follows: TP53I3 F: 5′-CCATGCAGGACTGAGTGGTG-3′, R: 5′-CTGCTCCAAGCTTTTCTGCC-3′; GAPDH F: 5′-CATGAGAAGTATGACAACAGCCT-3′, R: 5′-AGTCCTTCCACGATACCAAAGT-3′.

### Western blotting

Total proteins from H1299 and A549 cells were homogenized by using RIPA lysis buffer (Solarbio, Beijing, China). The protein sample (50 μg) was separated through the assay of sodium dodecyl sulfate–polyacrylamide gel electrophoresis and then transferred onto the polyvinylidene difluoride membrane (Millipore, USA). After blocked with 5% skim milk powder, the membrane was incubated with the primary antibodies (Bax, 1:1000, no. 50599–2-Ig; Bcl-2, 1:1000, no. 12789–1-AP; E-cadherin, 1:1000, no. 20874–1-AP; N-cadherin, 1:1000, no. 22018–1-AP; Vimentin, 1:500, no. 10366–1-AP; MMP-2, 1:1000, no. 10373–2-AP; MMP-9, 1:1000, no. 10375–2-AP; PI3K, 1:1000, no. 20584–1-AP; AKT, 1:1000, no. 10176–2-AP; mTOR, 1:500, no. 20657–1-AP; GAPDH, 1:1000, no. 10494–1-AP, Proteintech, USA. TP53I3, 1:500, no. ab96819; p-PI3K, 1:300, no. ab182651; p-AKT, 1:500, no. ab38449; p-mTOR, 1:500, no. ab109268, Abcam, USA) at 4 °C overnight. After that, the membrane was washed with Tris-buffered saline Tween-20 (TBST) and incubated with the secondary antibody (no. 7076, Cell Signal, USA) for 1 h. An enhanced chemiluminescence kit (ECL, Thermo Fisher, USA) was applied to determine the immunoreactive bands.

### Bioinformatics

The possible target genes of C3G were predicted though retrieving the Bioinformatics Analysis Tool for Molecular mechANism of Traditional Chinese Medicine (BATMAN-TCM, http://bionet.ncpsb.org.cn/batman-tcm/index.php/Home/Index/index), the genes with inference score > 20 in the Comparative Toxicogenomics Database (CTD) (http://ctdbase.org/) and the LUAD relative genes in the DisGeNET database (https://www.disgenet.org/). Then, the genes were intersected using Venn analysis (http://bioinformatics.psb.ugent.be/webtools/Venn/) [19]. Moreover, the differential expression of TP53I3 in LUAD patients tissues and the relative prognosis curve of LUAD patients were analyzed by the UALCAN (http://ualcan.path.uab.edu/) [20]. The database used in UALCAN was obtained from the Cancer Genome Atlas (TCGA) database (https://cancergenome.nih.gov/). The criteria for patients dividing into the two groups on prognosis curve is the Quartile.

### Statistical analysis

For statistical analysis, the GraphPad Prism 8.0 was used. The data are presented as mean ± standard deviation (SD). Comparisons among multiple groups were carried out by one-way analysis of variance (ANOVA) followed by a Bonferroni post hoc test. *P* < 0.05 was chosen as a statistically significant result. All experiments were performed in triplicate.

## Results

### C3G suppresses the viability of H1299 and A549 cells

**Fig. 1:**
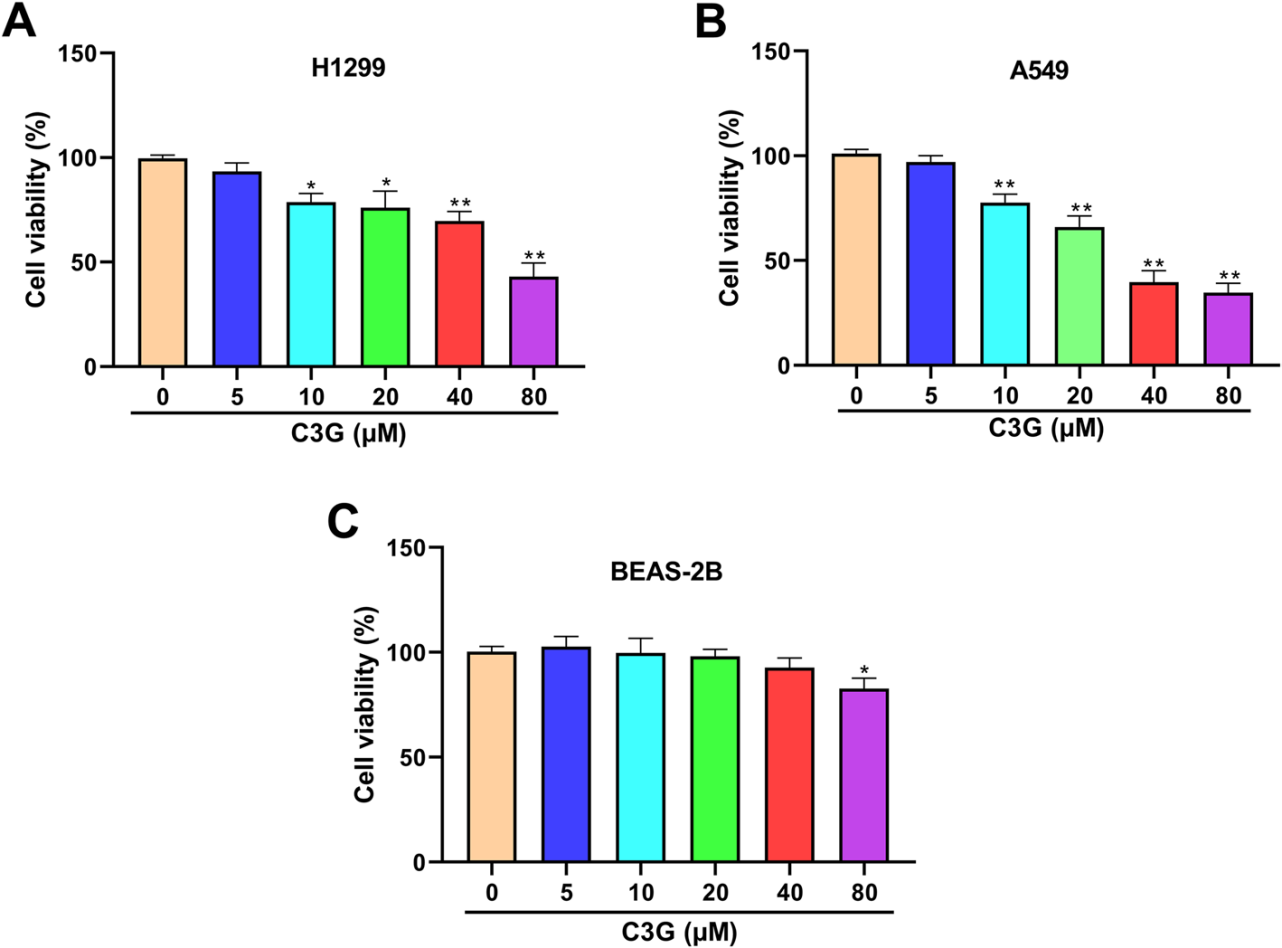
C3G suppressed the viability of H1299 and A549 cells. **A** The viability of H1299 cells followed by the treatment of C3G (0, 5, 10, 20, 40, and 80 μM) was detected by CCK-8 assay. **B** The viability of A549 cells followed by the treatment of C3G (0, 5, 10, 20, 40, and 80 μM) was detected by CCK-8 assay. **C** The viability of BEAS-2B cells followed by the treatment of C3G (0, 5, 10, 20, 40, and 80 μM) was detected by CCK-8 assay. ^*^P < 0.05,^**^P < 0.01, vs. C3G (0 μM) group

The effect of C3G on the viability of H1299, A549, and BEAS-2B cells was assessed by using the CCK-8 assay. As seen in Fig. 1A and B, cell viability was markedly reduced in C3G 10-, 20-, 40-, and 80-μM groups in both H1299 and A549 cells and in a dose-dependent manner (*P* < 0.05, *P* < 0.01). Moreover, only C3G 80 μM had little effect on normal lung epithelial cell BEAS-2B (*P* < 0.05, Fig. 1C). These results indicated that C3G may inhibit LUAD cell growth. In the subsequent experiments, C3G (10, 20, and 40 μM) was selected to further explore C3G effect on H1299 and A549 cell.

### C3G inhibits the proliferation and promotes the apoptosis in H1299 and A549 cells

**Fig. 2:**
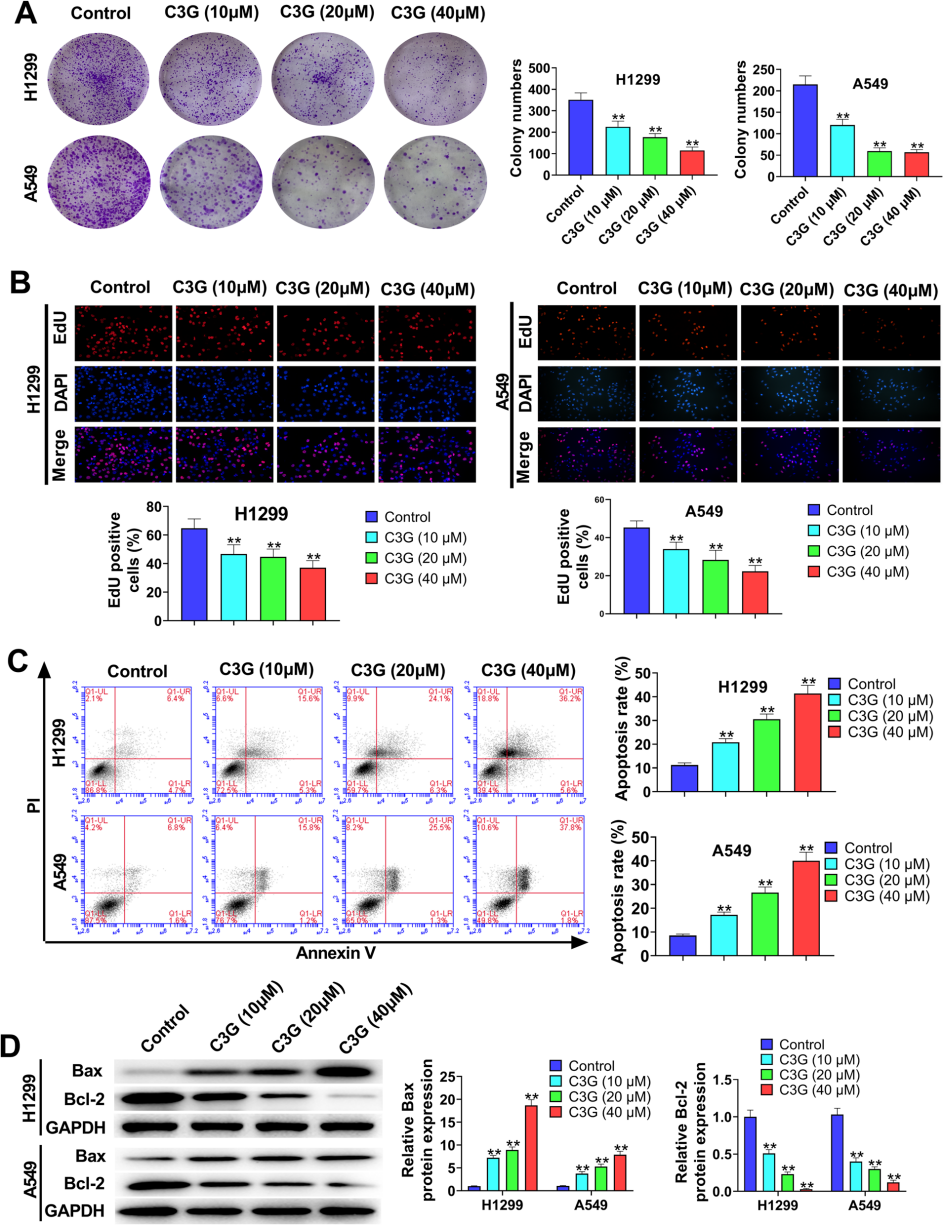
C3G inhibited the proliferation and promoted the apoptosis in H1299 and A549 cells. **A** After the treatment of C3G (10, 20, and 40 μM), the cell clones of H1299 and A549 cells were determined by colony formation assay. **B** After the treatment of C3G (10, 20, and 40 μM), the proliferation of H1299 and A549 cells was detected by using EdU assay. **C** After the treatment of C3G (10, 20, and 40 μM), the apoptosis of H1299 and A549 cells was detected by flow cytometry. **D** After the treatment of C3G (10, 20, and 40 μM), the expressions of Bax and Bcl-2 in H1299 and A549 cells were measured by western blot. ^**^P < 0.01, vs. Control group

The results of colony formation assay (Fig. 2A) and EdU assay (Fig. 2B) showed that C3G (10, 20, and 40 μM) notably inhibited the proliferation of H1299 and A549 (*P* < 0.01). As Fig. 2C showed, when compared with Control group, C3G (10, 20, and 40 μM) markedly facilitated the apoptosis of H1299 and A549 cells (*P* < 0.01). In addition, the function of C3G on the expressions of Bax and Bcl-2 was also investigated by western blot (Fig. 2D). The data revealed that the treatment of C3G (10, 20, and 40 μM) significantly increased the expression of Bax in H1299 and A549 cells (*P* < 0.01), but decreased Bcl-2 expression (*P* < 0.01). Altogether, the data demonstrated that C3G could inhibit the proliferation and promote the apoptosis in H1299 and A549 cells.

### C3G inhibits the migration and invasion in H1299 and A549 cells

**Fig. 3:**
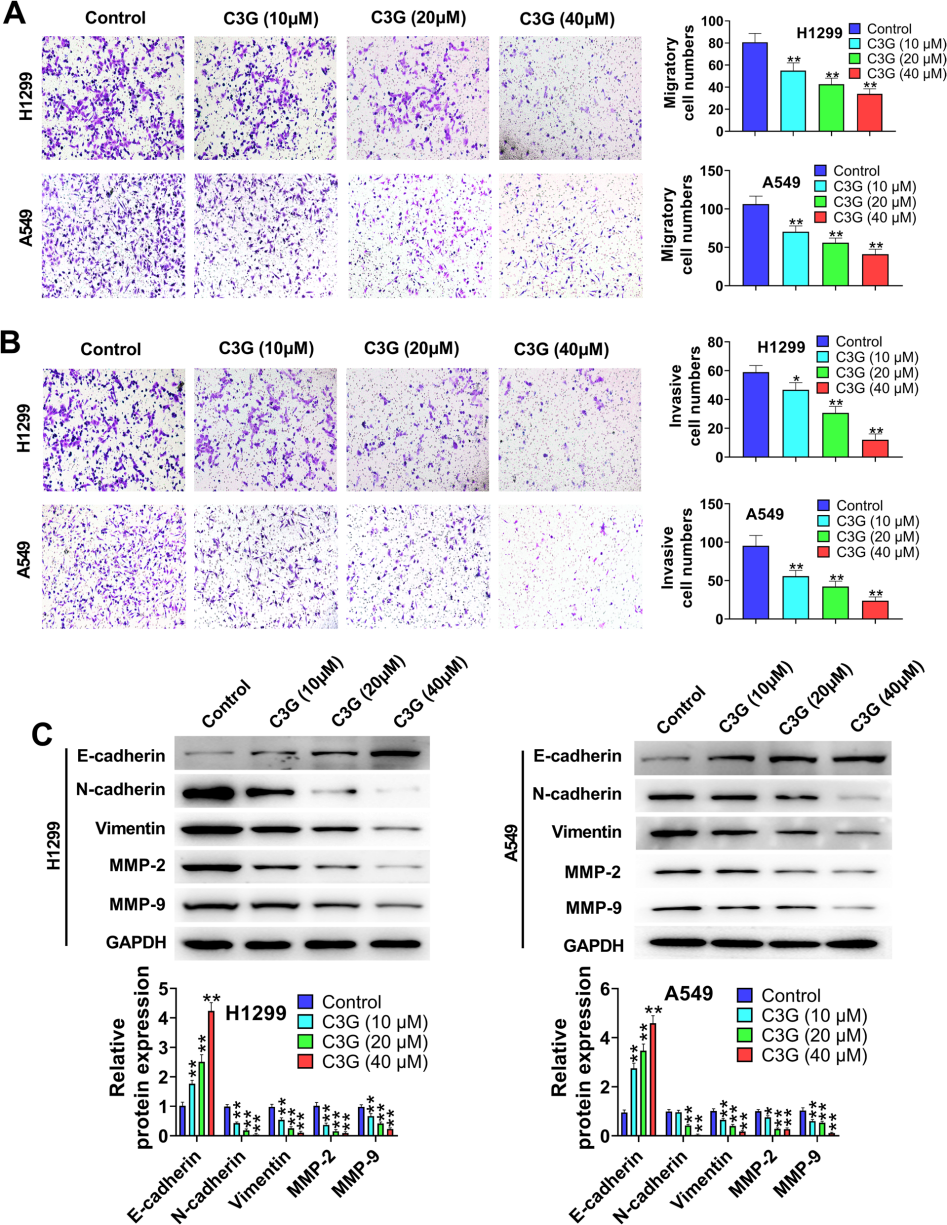
C3G inhibited the migration and invasion in H1299 and A549 cells. **A** The migration of H1299 and A549 cells followed by the treatment of C3G (10, 20, and 40 μM) was detected by transwell assay. **B** The invasion of H1299 and A549 cells followed by the treatment of C3G (10, 20, and 40 μM) was detected by transwell assay. **C** After the treatment of C3G (10, 20, and 40 μM), the expressions of E-cadherin, N-cadherin, Vimentin, MMP-2, and MMP-9 in H1299 and A549 cells were measured by western blot. ^*^P < 0.05, ^**^P < 0.01, vs. Control group

The migration and invasion in H1299 and A549 cells were assessed by transwell assay (Fig. 3A and B). The results showed that the treatment of C3G (10, 20, and 40 μM) significantly repressed the migration and invasion of H1299 and A549 cells (*P* < 0.05, *P* < 0.01) when compared with Control group. In addition, western blot results (Fig. 3C) demonstrated that C3G (10, 20, and 40 μM) markedly elevated E-cadherin expression level in H1299 and A549 cells (*P* < 0.01), but dramatically reduced the expressions of N-cadherin, Vimentin, MMP-2, and MMP-9 (*P* < 0.05, *P* < 0.01). All accumulating results indicated that C3G could inhibit the migration and invasion in H1299 and A549 cells.

### C3G suppresses the expression of TP53I3 in H1299 and A549 cells

**Fig. 4:**
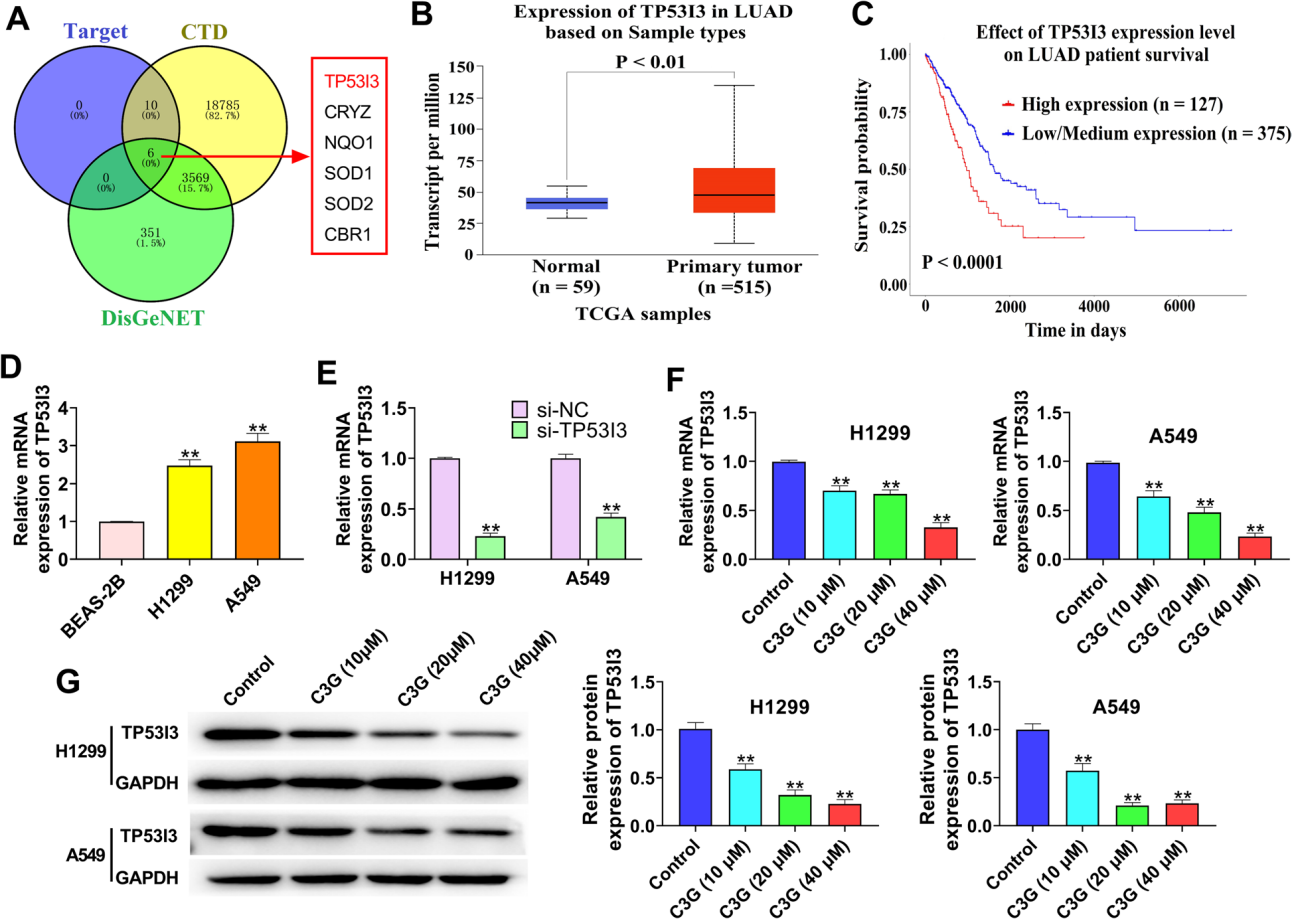
C3G suppressed the expression of TP53I3 in H1299 and A549 cells. **A** The database of BATMAN, CTD, and DisGeNET showed that C3G was closely associated with six genes (TP53I3, CRYZ, NQO1, SOD1, SOD2, and CBR1). **B** The expression of TP53I3 in LUAD tissues from TGCA. **C** Kaplan–Meier curve of the LUAD clinical outcome for TP53I3 from TCGA. **D** The expression of TP53I3 was detected by qRT-PCR in BEAS-2B, H1299, and A549 cells. **E** After transfection of si-TP53I3, the expression of TP53I3 in H1299 and A549 cells was detected by qRT-PCR. **F** After the treatment of C3G (10, 20, and 40 μM), the expression of TP53I3 in H1299 and A549 cells was detected by qRT-PCR. **G** After the treatment of C3G (10, 20, and 40 μM), the expression of TP53I3 in H1299 and A549 cells was measured by western blot. ^**^P < 0.01, vs. BEAS-2B cells group (**D**); ^**^P < 0.01, vs. si-NC group (**E**); ^**^P < 0.01, vs. Control group (**F** and **G**)

Based on the database of BATMAN, CTD, and DisGeNET, C3G was closely associated with six genes (TP53I3, CRYZ, NQO1, SOD1, SOD2, and CBR1) (Fig. 4A). In addition, the database of TCGA showed TP53I3 was highly expressed in LUAD tissues (Fig. 4B) and related to the prognosis of LUAD patients (Fig. 4C). Moreover, the upregulation of TP53I3 was also confirmed in LUAD cells (*P* < 0.01) (Fig. 4D). Thus, the relationship between C3G and TP53I3 was further investigated. As shown in Fig. 4E, qRT-PCR was used to verify the transfection efficiency. The results of qRT-PCR and western blot showed that the treatment of C3G (10, 20, and 40 μM) significantly inhibited TP53I3 expression in both H1299 and A549 cells (*P* < 0.01) (Fig. 4F and G). These results demonstrated that C3G could suppress the expression of TP53I3 in H1299 and A549 cells.

### C3G inhibits the proliferation, migration, and invasion, and promotes apoptosis in H1299 and A549 cells by downregulating TP53I3

**Fig. 5:**
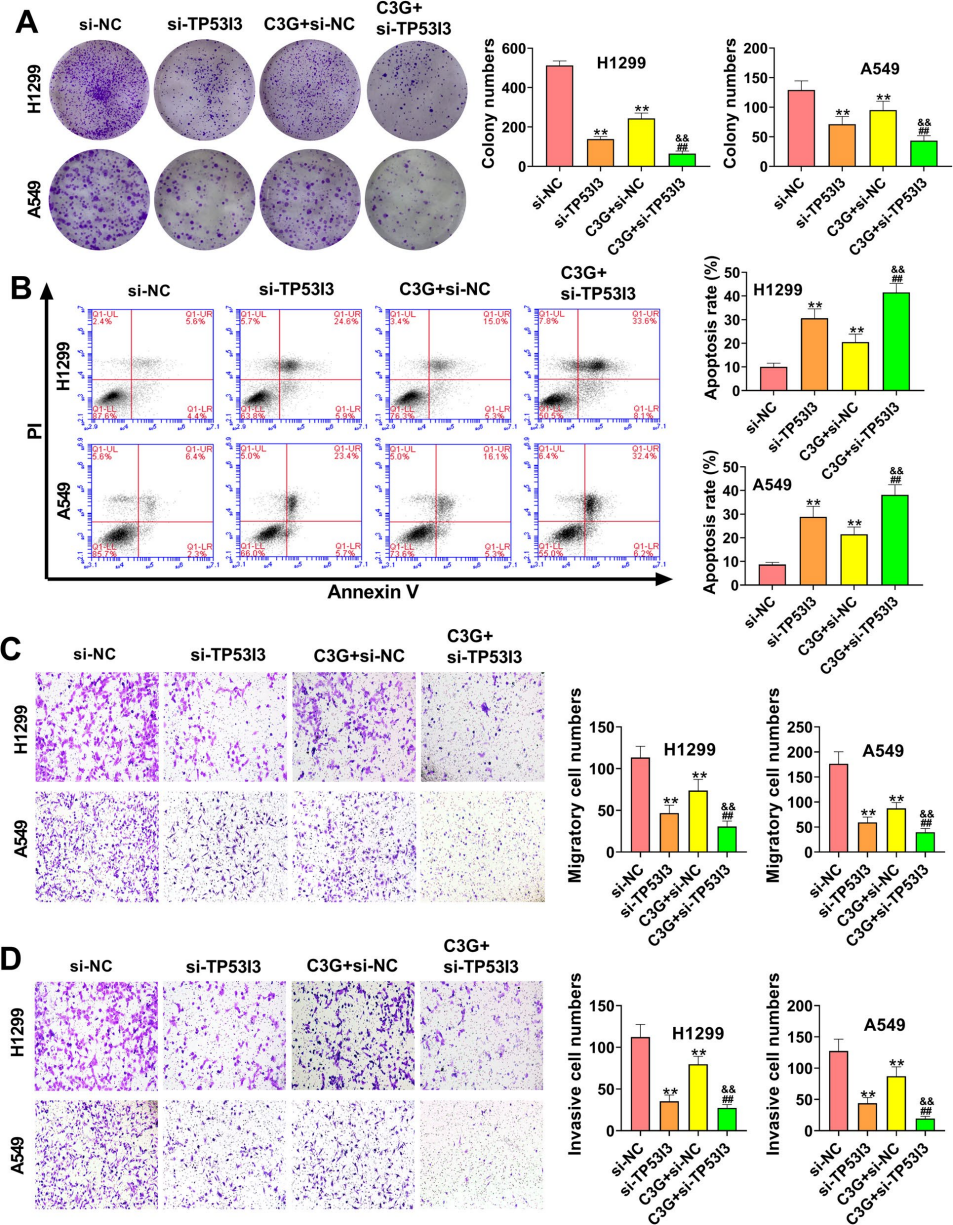
C3G inhibited the proliferation, migration, and invasion, and promoted apoptosis in H1299 and A549 cells by downregulating TP53I3. **A** After the treatment of C3G (40 μM) or si-TP53I3, the cell clones of H1299 and A549 cells were determined by colony formation assay. **B** After the treatment of C3G (40 μM) or si-TP53I3, the apoptosis of H1299 and A549 cells was detected by flow cytometry. **C** After the treatment of C3G (40 μM) or si-TP53I3, the migration of H1299 and A549 cells was detected by transwell assay. **D** After the treatment of C3G (40 μM) or si-TP53I3, the invasion of H1299 and A549 cells was detected by transwell assay. ^**^P < 0.01, vs. si-NC group; ^##^*P* < 0.01, vs. si-TP53I3 group; ^&&^*P* < 0.01, vs. C3G + si-NC group

As shown in Fig. 5A, the cell colonies of H1299 and A549 cells were significantly decreased in the si-TP53I3 and C3G + si-NC groups relative to the si-NC group (*P* < 0.01). When compared with the si-TP53I3 and C3G + si-NC groups, the cell colonies were markedly reduced in the C3G + si-TP53I3 group (*P* < 0.01) (Fig. 5A). The results of Fig, 5B showed that the apoptosis of H1299 and A549 cells was notably increased in the si-TP53I3 and C3G + si-NC groups compared with the si-NC group (*P* < 0.01). Meanwhile, in comparison with the si-TP53I3 and C3G + si-NC groups, cell apoptosis was significantly elevated in the C3G + si-TP53I3 group (*P* < 0.01) (Fig. 5B). Additionally, transwell assay confirmed that the migration and invasion of H1299 and A549 cells were significantly increased in the si-NC group relative to the si-TP53I3 and C3G + si-NC groups (*P* < 0.01), but dramatically decreased in the C3G + TP53I3 group (*P* < 0.01) (Fig. 5C and D). These results indicated that C3G could inhibit the proliferation, migration and invasion, and promote apoptosis in H1299 and A549 cells through downregulating TP53I3.

### C3G inhibits the activation of the PI3K/AKT/mTOR pathway in H1299 and A549 cells by downregulating TP53I3

**Fig. 6:**
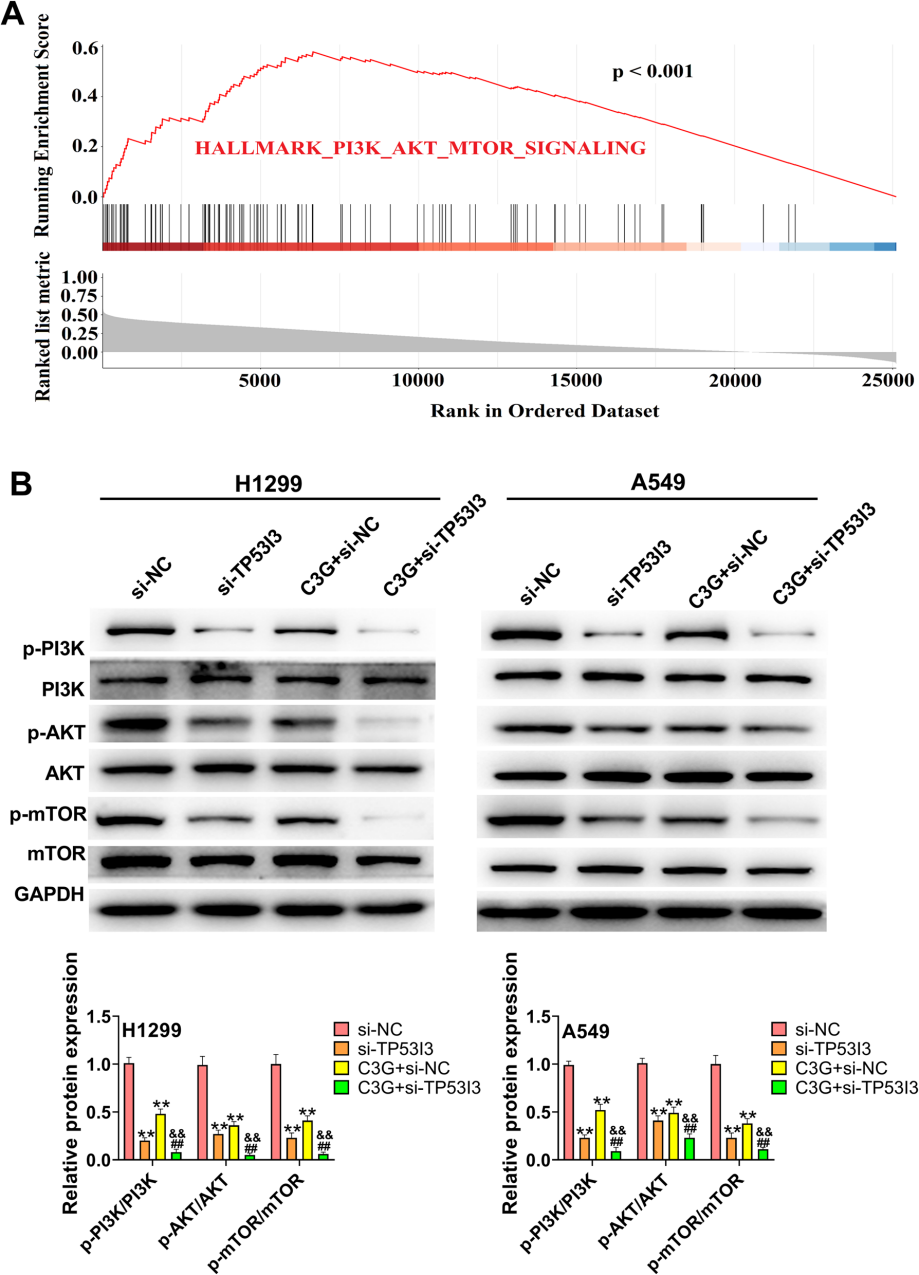
C3G inhibited the activation of PI3K/AKT/mTOR pathway in H1299 and A549 cells by downregulating TP53I3. **A** GSEA showed that the expression of TP53I3 was positively correlated with PI3K/AKT/mTOR pathway. **B** After the treatment of C3G (40 μM) or si-TP53I3, the expressions of p-PI3K, PI3K, p-AKT, AKT, p-mTOR, and mTOR in H1299 and A549 cells were measured by western blot. ^**^P < 0.01, vs. si-NC group; ^##^*P* < 0.01, vs. si-TP53I3 group; ^&&^*P* < 0.01, vs. C3G + si-NC group

As seen in Fig. 6A, the gene set enrichment analysis (GSEA) showed that the high expression of TP53I3 was positively associated with the “PI3K/AKT/mTOR pathway” in LUAD. Therefore, whether C3G could regulate PI3K/AKT/mTOR pathway via downregulating TP53I3 was further assessed. Western blot results showed that the expressions of p-PI3K, p-AKT, and p-mTOR in H1299 and A549 cells were markedly lower in the si-TP53I3 and C3G + si-NC groups than that in the si-NC group (*P* < 0.01) (Fig. 6B). On the contrary, when compared with the si-TP53I3 and C3G + si-NC groups, the expressions of p-PI3K, p-AKT, and p-mTOR in H1299 and A549 cells were significantly reduced in the C3G + TP53I3 group (*P* < 0.01) (Fig. 6B). All these results confirmed that C3G could inhibit the activation of the PI3K/AKT/mTOR pathway in H1299 and A549 cells by downregulating TP53I3.

## Discussion

The mortality of lung cancer ranks first among all kinds of malignant tumors [21]. Despite encouraging progress with new drugs in LUAD, the prognosis of patients with advanced cancer is still poor. Therefore, the exploration of novel drugs and therapeutic targets for LUAD treatment is urgently needed. In this study, C3G could inhibit the proliferation, migration, and invasion, and also facilitate the apoptosis through downregulating TP53I3 and inhibiting the PI3K/AKT/mTOR pathway in LUAD.

A growing number of evidence has confirmed that C3G plays an important role in a variety of cancers. For instance, C3G is able to suppress tumor cell growth, migration, and invasion, and induce apoptosis in breast cancer [22]. Hosseini et al. have indicated that C3G has a significantly pro-apoptotic effect in the glioblastoma cells [23]. In addition, the study by Chen et al. [12] has reported that C3G can exhibit an inhibitory effect on the migration and invasion of lung cancer cells. In the present study, C3G was also confirmed to inhibit the proliferation, migration, and invasion, and promote the apoptosis of LUAD cells. The epithelial–mesenchymal transition (EMT) status plays a crucial role in cancer cell metastasis and invasion [24]. EMT is accompanied by the downregulation of epithelial markers, such as E-cadherin, and the upregulation of mesenchymal markers such as N-cadherin and Vimentin [25, 26]. The results confirmed that C3G significantly increased E-cadherin expression, but decreased the expression of N-cadherin and Vimentin, suggesting that C3G could inhibit LUAD cell migration and invasion by repressing EMT process.

In recent years, several studies have suggested that TP53I3 may play an important role in various types of cancers. TP53I3 is reported to be highly expressed in papillary thyroid carcinoma and plays oncogenic roles through the activation of the PI3K/Akt/PTEN pathway [16]. The database of TCGA showed TP53I3 was highly expressed in LUAD tissues and correlated with the poor prognosis of LUAD patients, further confirming that TP53I3 may be an oncogenic gene in LUAD. However, whether C3G is able to regulate TP53I3 in LUAD remains unknown. Thus, the relationship between C3G and its target genes was further explored via the database of BATMAN, CTD, and DisGeNET. The results showed that C3G was closely associated with six genes (TP53I3, CRYZ, NQO1, SOD1, SOD2, and CBR1). Moreover, C3G was found to significantly inhibit the expression of TP53I3 in H1299 and A549 cells. More and more studies have shown that TP53I3 can exhibit important effects in cancer cell biological behaviors. For example, TP53I3 overexpression could elevate the colony formation, migration, and invasion ability of colon cancer cells [17]. Xu et al. have reported that TP53I3 could facilitate the growth of papillary thyroid cancer through activating the PI3K/AKT/PTEN pathway [16]. Gu et al. have confirmed that TP53I3 could promote cell migration and invasion in lung adenocarcinoma [18]. In the present study, TP53I3 silencing significantly inhibited the proliferation, migration, and invasion, and promoted apoptosis in LUAD cells. Additionally, C3G could also inhibit the proliferation, migration, and invasion, and promote apoptosis in H1299 and A549 cells through downregulating TP53I3.

Emerging evidence has suggested that the PI3K/AKT/mTOR pathway is a signal transduction pathway involved in the modulation of multiple cellular functions including cell survival, proliferation, cell cycle, apoptosis, autophagy, differentiation, migration, and invasion [27–29]. In recent years, more and more researches have reported that the PI3K/AKT/mTOR pathway is aberrantly activated in NSCLC and associated with the progression of NSCLC [30–33]. A study by Xu et al. [16] has reported that TP53I3 could play oncogenic roles in papillary thyroid cancer through the activation of the PI3K/AKT/PTEN pathway. GSEA data suggested that the high expression of TP53I3 was positively associated with the “PI3K/AKT/mTOR pathway” in LUAD. In the present study, C3G could inhibit the activation of PI3K/AKT/mTOR pathway by downregulating TP53I3 in LUAD cells.

In conclusion, C3G could inhibit the proliferation, migration, and invasion, and also facilitate the apoptosis through downregulating TP53I3 and inhibiting PI3K/AKT/mTOR pathway in LUAD. These findings implied that C3G may be an effective therapeutic drug for LUAD treatment.

## Data Availability

The datasets used and analyzed during the current study are available from the corresponding author on reasonable request.
